# Spherical rotary cell seeding system for production of small-caliber tissue-engineered blood vessels with complex geometry

**DOI:** 10.1038/s41598-023-29825-0

**Published:** 2023-02-21

**Authors:** Alyssa Brodeur, Alexandre Winter, Vincent Roy, Lydia Touzel Deschênes, François Gros-Louis, Jean Ruel

**Affiliations:** 1 Department of Surgery, Faculty of Medicine, Laval University, Quebec City, QC Canada; 2 Division of Regenerative Medicine, CHU de Quebec Research Center, Laval University, Quebec City, QC Canada; 3 Department of Mechanical Engineering, Faculty of Sciences and Engineering, Laval University, Quebec City, QC Canada

**Keywords:** Tissues, Tissue engineering, Cardiovascular models

## Abstract

Entirely biological human tissue-engineered blood vessels (TEBV) were previously developed for clinical use. Tissue-engineered models have also proven to be valuable tools in disease modelling. Moreover, there is a need for complex geometry TEBV for study of multifactorial vascular pathologies, such as intracranial aneurysms. The main goal of the work reported in this article was to produce an entirely human branched small-caliber TEBV. The use of a novel spherical rotary cell seeding system allows effective and uniform dynamic cell seeding for a viable in vitro tissue-engineered model. In this report, the design and fabrication of an innovative seeding system with random spherical 360° rotation is described. Custom made seeding chambers are placed inside the system and hold Y-shaped polyethylene terephthalate glycol (PETG) scaffolds. The seeding conditions, such as cell concentration, seeding speed and incubation time were optimized via count of cells adhered on the PETG scaffolds. This spheric seeding method was compared to other approaches, such as dynamic and static seeding, and clearly shows uniform cell distribution on PETG scaffolds. With this simple to use spherical system, fully biological branched TEBV constructs were also produced by seeding human fibroblasts directly on custom-made complex geometry PETG mandrels. The production of patient-derived small-caliber TEBVs with complex geometry and optimized cellular distribution all along the vascular reconstructed may be an innovative way to model various vascular diseases such as intracranial aneurysms.

## Introduction

The advancement of tissue engineered vascular grafts in recent years presents a promising clinical option for the treatment of vascular diseases or to provide alternative in vitro models to study these complex disorders^1,2^. Through model refinement, it is now possible to produce patient-derived tissue-engineered blood vessels (TEBV) with defined genetic backgrounds to better understand the pathobiology behind vascular diseases^3,4^. Different techniques to generate TEBVs have been developed over the years, each showcasing pros and cons, and can be classified in three main categories: (1) vascular conduits made of cells seeded on manufactured scaffolds, (2) vascular conduits made by cell-sheet engineering and (3) bioprinting^5,6^. One of the challenges in vascular tissue engineering is still, however, to improve cellular seeding, distribution, and organization to homogeneously incorporate cells onto a tubular structure. Consequently, dynamic cell seeding techniques have established themselves over simpler static approaches^7–9^. Furthermore, in a tri-dimensional (3D) environment, a uniformly monitored cell distribution is needed to foster homogeneous tissue remodeling and to avoid competition for nutrients in areas with higher cell densities^10–13^. The current state of dynamic cell seeding allows an easy production of linear TEBV with the use of roll bottles and the perfused seeding of endothelial cells in a tubular construct. However, these are not ideal for production of more complex geometry tri-layered TEBV composed of an *adventitia*, a *media* and an *intima tunica*^4,14–16^.

Previously, the production of self-assembled linear small-caliber blood vessels seeded on polyethylene terephthalate glycol (PETG) pretreated with ultraviolet-C rays (UV-C) has been shown to ensure proper cellular attachment and optimized extracellular matrix (ECM) secretion/assembly^14^. To produce TEBVs with complex geometry and to improve cell seeding along the scaffolds, we developed a rotary system with a random rotation movement allowing effective and uniform cell distribution. We describe here the design and fabrication of an innovative rotary seeding system able to perform full 360° rotation and produce completely biological branched tissue-engineered vessel *adventitia* (TEBV-A)*.*

## Results

### Design, conception and fabrication of a novel seeding system

**Figure 1:**
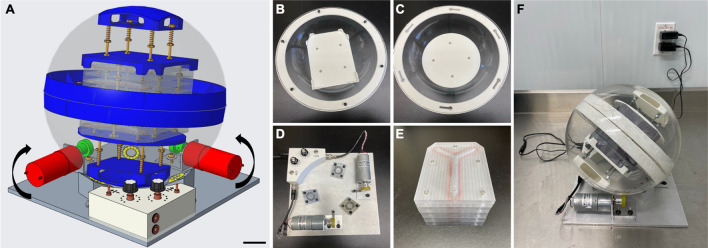
Rotary seeding system. (**A**) Computer-aided design (CAD) of the system with CREO 5.0 software with directional indicators; (**B,C**) photographs of the male and female halves of the acrylic sphere with 3D-printed closure ring and plates to keep the seeding chambers in place; (**D**) aluminum support plate with three ball bearing-type supports, two motors and an electronic control unit; (**E**) five custom-made acrylic cell seeding chambers; (**F**) assembled rotary seeding system. Scale bar = 5 cm.

The initial specifications for the conception of this novel rotary seeding system were a 360° rotation with adjustable speed, an operation time of at least 24 h, and a production capacity of a maximum of five TEBVs simultaneously. The design was made to be simple, straightforward, and easy-to-use (Fig. 1A). This model includes an acrylic sphere made from two halves put into a spherical rotational movement by two motors on a support plate so that TEBVs can be produced inside the seeding chambers held in the middle of the sphere (Fig. 1B–E). This seeding system has been adapted to have a 360° random rotation. The rotation is considered to be random because the constant speed of the motors is used all along the seeding time and the imperfections in the sphere shape cause changes in the movement axis (Supplementary Video 1). The system also has an adjustable speed of 63 to 135°/minute, an operation time of more than 24 h, and a capacity production of five TEBVs (Fig. 1F). It can also be placed into an environment with a controllable temperature for the duration of the seeding step.

**Figure 2:**
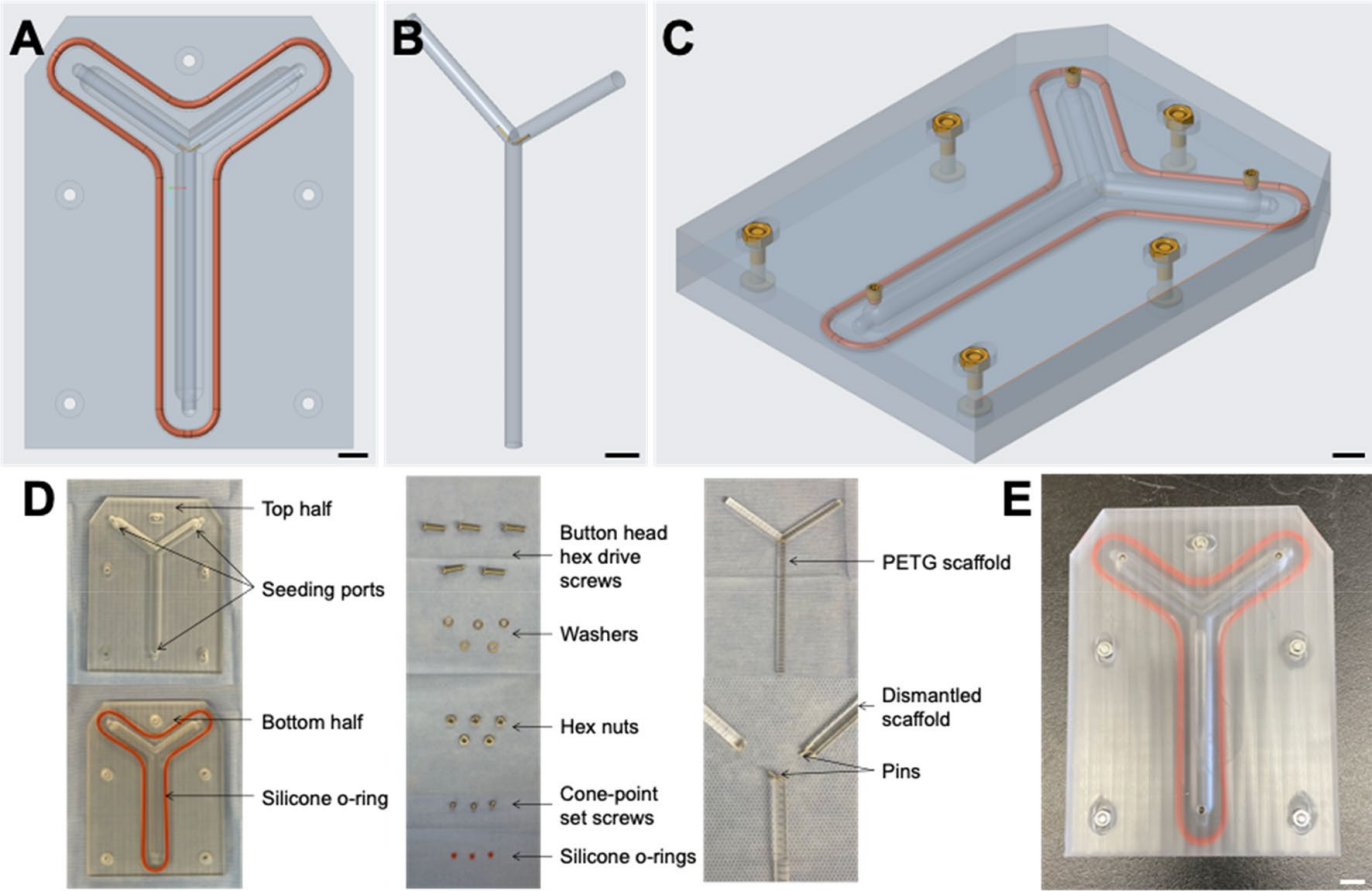
Custom made branched seeding chambers. (**A**) CAD of the system with CREO 5.0 software of the bottom half acrylic seeding chamber with Y-shaped indent and Y-shaped o-ring; (**B**) CAD of custom Y-shaped PETG scaffold with stainless-steel dowel pins; (**C**) CAD of the complete seeding chamber closed with hardware and view of small screws for seeding ports; (**D**) photograph of all the parts and hardware of the seeding chambers inside the biological hood on a sterile field; (**E**) photograph of the complete chamber closed with hardware and view of small screws for the seeding port. Scale bar = 1 cm.

Custom-made seeding chambers were designed to seed human fibroblast cells on branched PETG scaffolds (Fig. 2). The chambers are composed of two polycarbonate halves with custom-machined Y-shaped indents suited to house Y-shaped 4.8 mm diameter PETG scaffolds (Fig. 2A–C). These scaffolds are made from three separate parts held together by stainless steel pins so that the different branches can be removed for easy disassembly. The bottom half has a Y-shaped o-ring, to make the chamber watertight and the top half, has three small openings allowing to add cells and culture media (Fig. 2A). The two halves are held together with stainless steel hardware. All parts used for the chambers can be sterilized by autoclaving and then used under a biological hood using aseptic technique to seed human cells (Fig. 2D). Five of the closed seeding chambers are needed to be placed inside the rotary system so the sphere can function properly (Fig. 2E).

### Optimal cell seeding parameters

**Figure 3:**
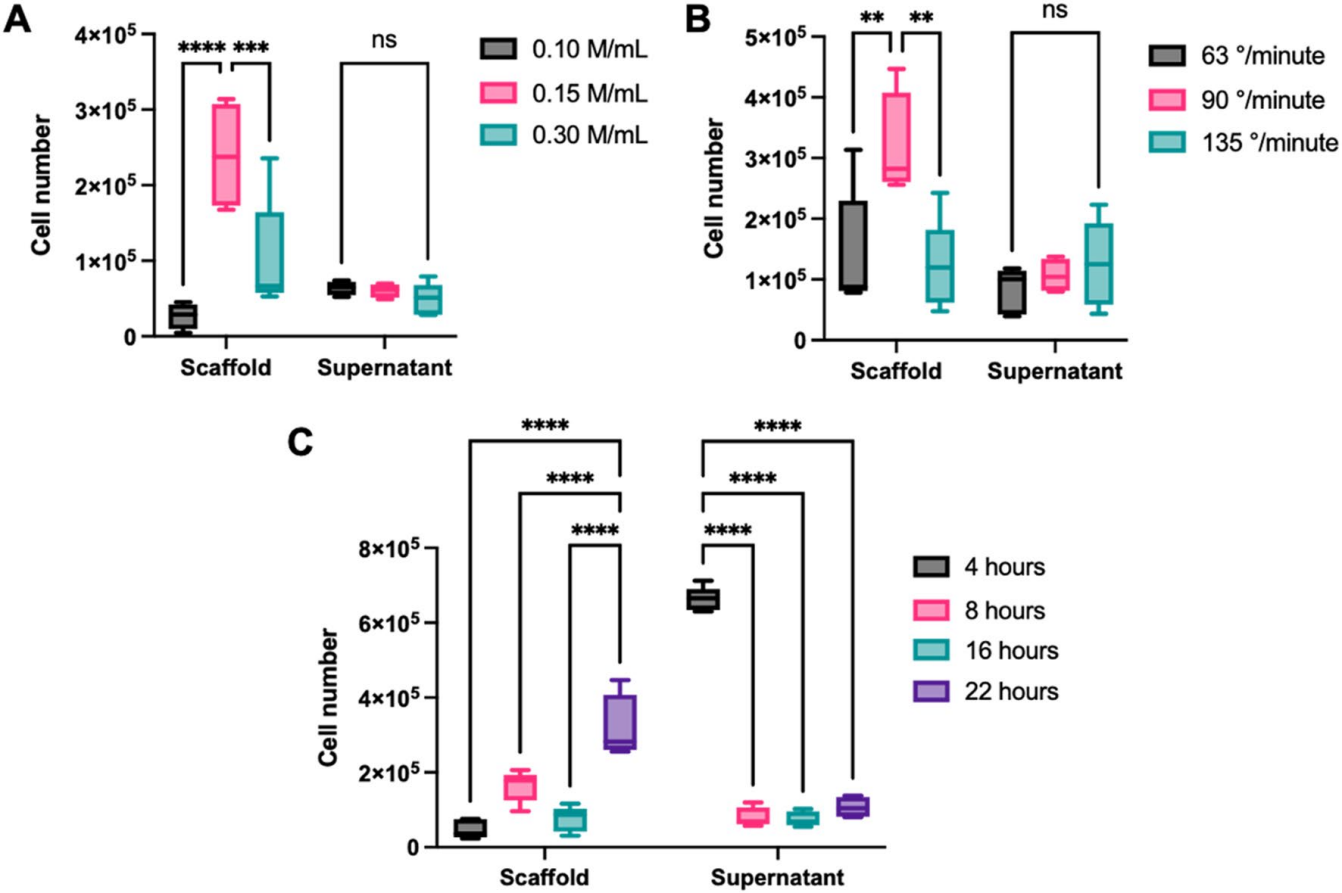
Number of cells adhered on the scaffolds and in the supernatant depending on cell concentration, speed of seeding system and incubation time. (**A**) Three cell concentrations in million cells per millilitre of medium (M/mL) were evaluated by seeding cells at 90°/minute for 22 h; (**B**) three system speeds (°/min) were assessed by seeding 0.15 M/mL cells for 22 h; (**C**) four incubation times (hours) were analyzed by seeding 0.15 M/mL at 90°/min. For statistical analyses, a two-way ANOVA with Tukey’s multiple comparison test was performed. Graph (**A–C**) show box and whiskers with max and min. *n* = 4–5/group. *P < 0.05, **P < 0.01, ***P < 0.001 and ****P < 0.0001. *ns* non-significative.

To determine the optimal cell seeding parameters, we first tested different cellular concentrations. Three initial cellular concentrations, indicated here in million cells per millilitre of culture medium (M/mL), were used (0.1 M/mL, 0.15 M/mL et 0.30 M/mL) to seed the chambers. Cells were allowed to adhere to a 4.8 mm diameter PETG mandrel for a 22-h period at a medium speed of 90°/min. Cell-seeded mandrels were then incubated with trypsin for 10 min and the detached cells were counted. A cell concentration of 0.15 M/mL was found to be the best cell seeding parameter (0.15 M/mL compared to 0.1 M, *P* < 0.0010; 0.15 M/mL compared to 0.3 M/mL, *P* = 0.3805) (Fig. 3A). No significant difference was observed between conditions for the number of cells in the supernatant after seeding (Fig. 3A). Using this optimal cell concentration, we then wanted to determine the best seeding speed, i.e., the optimal speed to set up the spherical rotating system allowing the most significant number of cells to adhere to the scaffold. Three different seeding speeds were tested (63°/min, 90°/min and 135°/min). The number of cells adhered to the PETG scaffold was found to be significantly higher when using the midrange 90°/min seeding speed (63°/min compared to 90°/min; *P* = 0.0321, and 90°/min compared to 135°/min; *P* = 0.0119) (Fig. 3B). No significant difference was observed for the number of cells in the supernatant after cell seeding (Fig. 3B). Different incubation times were also tested following cell seeding within the chambers (4 h, 8 h, 16 h and 22 h). An incubation time of 22 h was found to be the best parameter here as more cells adhered to the scaffold over any other tested incubation time (*P* < 0.0001). In addition, more cells were counted in the supernatant after 4 h of seeding compared to any other time (*P* < 0.0001) indicating that cells did not have the time to properly adhere to the scaffold and were still in the culture medium (Fig. 3C). Overall, the optimal cell seeding parameters were 0.15 M/mL cells at a speed of 90°/min for 22 h at 37 °C.

### Comparison between the novel spherical seeding method to other techniques

**Figure 4:**
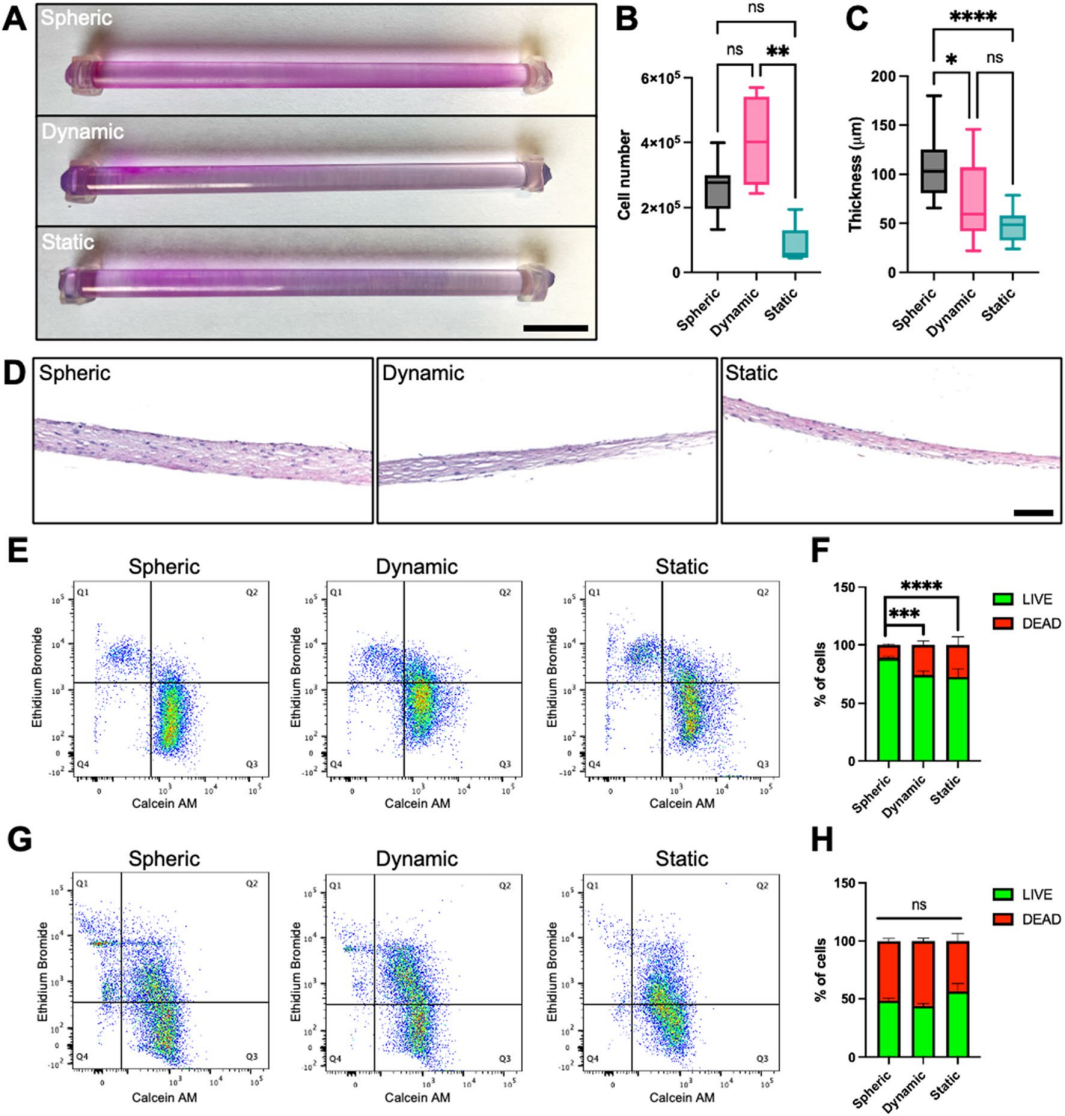
Cellular distribution and viability in post-seeded and matured TEBV-A produced by different seeding techniques. (**A**) Photograph of Rhodanile Blue stained cells on PETG scaffold post-seeding. Scale bar = 1 cm; (**B**) number of cells adhered to the scaffold following a 22-h seeding period; (**C**) TEBV-A tissue thicknesses (µm) measured after a 42 day maturation period; (**D**) Hematoxylin and eosin (H&E) staining of histological sections collected from matured TEBV-A produced using different seeding techniques (spheric, dynamic and static). Scale bar = 100 µm; (**E,F**) Live/dead assay on post-seeded TEBV-A harvested cells analyzed by flow cytometry; (**G,H**) Live/dead assay on matured TEBV-A harvested cells. (**B**,**C)** Box and whiskers with max and min. For statistical analyses, a Kruskal–Wallis test with Dunn’s multiple comparison test was performed. *n* = 4–24. (**F,H)** Stacked bars with standard deviation. For statistical analyses, a two-way ANOVA with Tukey’s multiple comparison test was performed. *n* = 5. *P < 0.05, **P < 0.01, ***P < 0.001 and ****P < 0.0001. *ns* non-significative.

The novel spherical seeding technique was compared to a dynamic and a static seeding method. First, cell distribution on PETG scaffolds post-seeding can be observed with a Rhodanile Blue staining (Fig. 4A). Apparent uniform cell distributions can be observed on the mandrels using the spheric technique when compared to other seeding techniques (Fig. 4A and Supplementary Fig. 1). Furthermore, no statistically significant difference between spherical seeding and the other techniques was measured following proper counts of cells adhered to the scaffolds after the 22-h seeding period (Fig. 4B). Remarkably, TEBV-A, produced using the described spherical system, were statistically ticker over TEBV-A produced using the other seeding techniques after a maturation period of 42 days of culture following initial seeding (*P* < 0.05 and *P* < 0.0001) (Fig. 4C,D), indicating that a uniform distribution may be important for the production of thicker TEBV-A.

Another important aspect to consider here and to compare between different seeding methods is cellular viability. Therefore, flow cytometry live/dead assays were used to quantify cellular viability following the 22-h seeding (post-seeding) and 42 days maturation periods. Post-seeding viability was found to be significantly higher while using the spherical seeding system compared to other seeding methods; dynamic (*P* < 0.001) and static (*P* < 0.001) (Fig. 4E,F and Supplementary Fig. 2A). Although considerably lower, no difference in cell viability was measured, however, following the 42 days post-seeding maturation period in any of the tested seeding techniques (Fig. 4G,H and Supplementary Fig. 2B). The lower cellular viability measured in matured TEBV-A could simply be explained here by the 30-min enzymatic digestion needed to harvest the cells from the ECM for flow cytometry analysis; this step is not needed in post-seeding period. Overall, a more uniform cellular distribution, a thicker TEBV-A, as well as an enhanced cell viability were measured using the developed spherical rotary system when compared to other dynamic and static seeding techniques.

### Characterization of produced tissue-engineered branched blood vessel—*adventitia*

**Figure 5:**
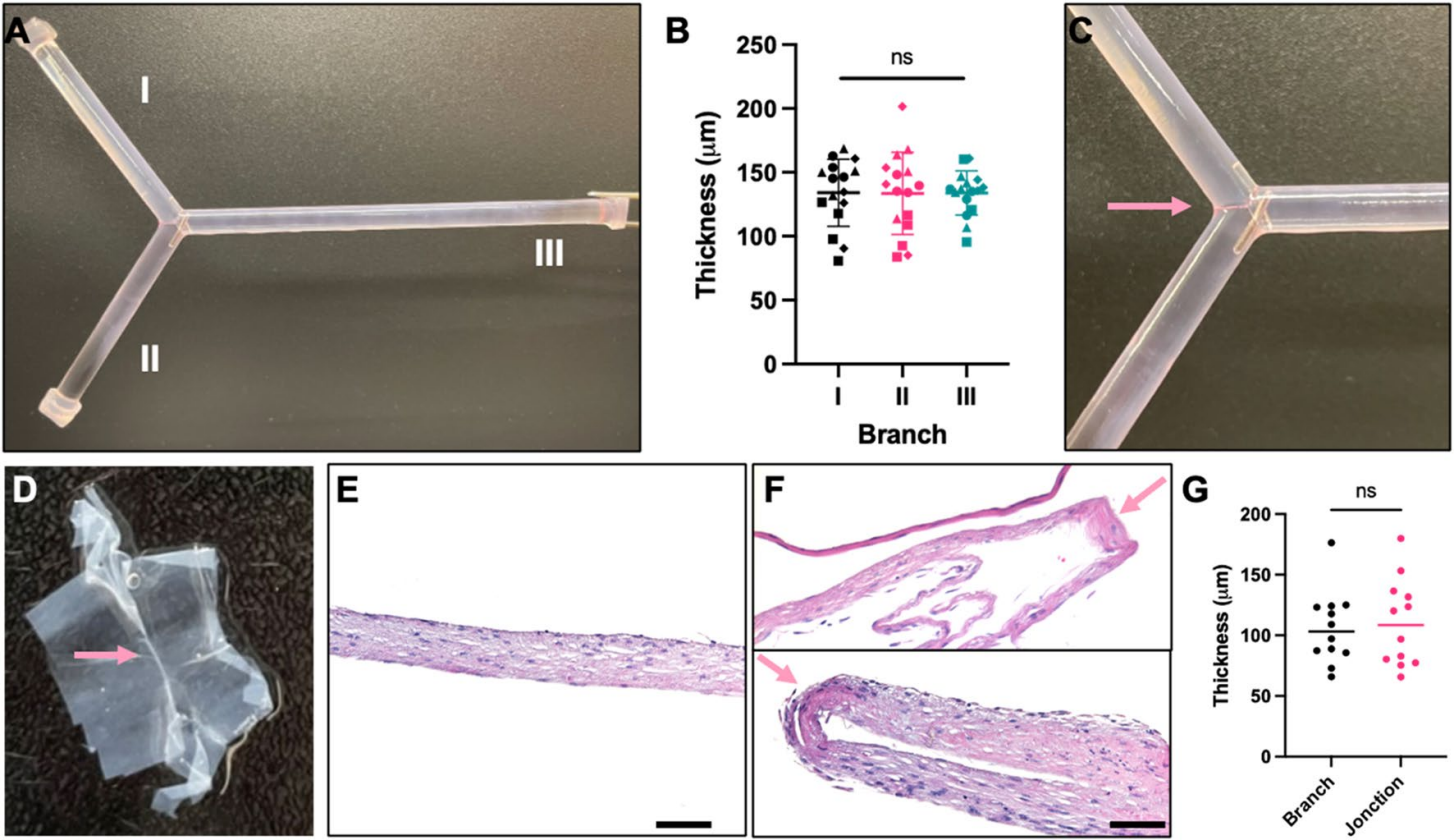
Macroscopic and microscopic characterization of the branched TEBV-A produced with the developed rotary seeding system. (**A**) Photograph of a “Y” geometry TEBV-A on a branched PETG scaffold; (**B**) tissue thickness of the three branches (I, II, III) of TEBV-A in µm. *N* = 4, *n* = 4; (**C**) close-up photograph of TEBV-A junction on the scaffold (**D**) and cut off from the scaffold; (**E**) H&E staining of the branch and (**F**) junction of TEBV-A sample cut off from the scaffold; (**G**) Tissue thickness of histological sections of the branches and the junction of TEBV-A in µm. *n* = 12. Arrows show junction site. For statistical analyses, a Kruskal–Wallis test with Dunn’s multiple comparison test was performed for graph (**B)** and a Welch’s t-test for graph (**G**). Graph (**B,G**) shows scatter plot with bars and standard deviation. Scale bar = 100 µm. *ns* non-significative.

Fully biological branched TEBV-A, made with human fibroblasts, were produced using the predetermined optimal cell seeding parameters. Following a cell seeding incubation period of 22 h, the seeding chambers were disassembled from the system. The cell-seeded scaffolds were then placed in culture plates and were maintained in culture for 42 days. Tissue thicknesses were measured using a laser micrometer on each branch (Fig. 5A). No statistically significant difference was found for all three branches of the TEBVs, indicative of uniform cell distribution all along the seeded mandrels (Fig. 5A,B). To produce TEBVs with complex geometry such as Y-shaped scaffolds, it was indeed crucial to design a cell seeding system enabling uniform cell distribution all along the scaffolds and particularly at the branched/junction site. The branched TEBV-A all showed uniformly distributed cells at the critical junction site macroscopically (Fig. 5C,D). In addition, tissue thicknesses were measured from histological cross-sections of the branches and the junctions (Fig. 5E,F) and no statistically significant differences were found (Fig. 5G).

## Discussion

In this article, we succeeded in designing and building a rotating spherical cell seeding system able to produce biological small-caliber TEBVs with complex geometry, entirely made of human cells. Cell seeding parameters were optimized to improve cellular distribution and adherence all along scaffold areas, even at the critical branched/junction point of the designed proof-of-concept “Y” shaped TEBVs. Compared to other dynamic and static seeding approaches, our novel spherical seeding method allows the cells to be more uniformly distributed all along the produced TEBV, demonstrated higher post-seeding cellular viability and produced thicker tissues. The described system included custom-machined and easily sterilizable seeding chambers containing UV-C treated PETG scaffolds designed to fit within the seeding chambers. Noteworthy, seeding chambers can easily be redesigned with other geometries/specifications to adjust for different blood vessel shape, size and angle at the bifurcation.

A cell concentration of 0.15 M/mL during the initial seeding of the chambers, combined to a random rotation in all directions (x, y and z) for 22 h at a speed of 90°/min were found to be the optimal seeding parameters to favour cell adherence and distribution along the PETG scaffold. Following the 22-h incubation period within the seeding chambers, the cell-seeded scaffolds were removed from the chambers and cultured for 42 days in culture medium to promote ECM production, assembly and maturation of TEBVs. Kinetics of the interactions between cells and solid scaffolds followed the Langmuir model, for which its three assumptions were adapted to cell culture: (i) cells cannot attach to form more than a monolayer; (ii) cells can attach to all surfaces of the scaffold; (iii) cells can adhere to the scaffold independently of cells already attached until a monolayer is reached^17,18^. The UV-C treatment of PETG is known to promote cell adherence by modifying the plastic’s carbonyl groups, which in turn augment the hydrophilic properties of the material^14,19–21^. In accordance with the Langmuir theory, the rotational movement of the described dynamic seeding system facilitates cell-scaffold interactions on the entire surface of the treated plastic mandrel, allowed for better cell adherence and distribution^7,8,22^. Indeed, the 360° random movement of this novel system allowed for cells to encounter all parts of the complex geometry scaffold during the seeding period. This was indicated by the uniform tissue thicknesses measured along the branches of the “Y” shaped TEBV. Langmuir’s third assumption implies that once a monolayer is reached, cells stop attaching independently to the scaffold and start to interact with other attached cells. This increase in cell–cell interactions over time may have promoted the cells to detach from the scaffold and explain why equal or fewer cells were retrieved from PETG with a higher cell density, effectively reaching a plateau^17,21^.

The system’s median speed was considered optimal for cell seeding at 90°/min. While all tested speeds were relatively low, the system needed to be fast enough to keep the cells suspended in the medium for the entire duration of seeding, but slow enough for the cells to properly interact and attach to the plastic. A longer seeding period of 22 h was also required to increase cellular attachment. Slow speeds for longer periods of time have also been shown to promote better cell attachment in other models^23,24^. Cell seeding parameters, such as speed, cell density and time, being dependent on cellular type (cell-scaffold interaction, cell adherence, matrix production capacity), therefore need to be optimized for each tissue-engineered model and type of cell to be seeded^9,25–28^. We estimated the sedimentation velocity (V) within the seeding chambers of 2.5 µm/s for the tested fibroblast population, using the Stokes’s Law in fluid mechanics V = (g(ρ_p_ − ρ_f_)d^2^)/18μ calculated using the following constants: (gravity (g: 9.81 m/s^2^), cell density (ρ_p_: 1050 kg/m^3^), Dulbecco’s Modified Eagle Medium (DMEM) density (ρ_f_: 1007 kg/m^3^), fibroblast cell diameter (d: 10 µm) and DMEM viscosity (μ: 0.00093 Pa s)^29,30^. Although slow, this sedimentation rate of fibroblasts combined with the random rotation movement of the sphere for 22 h enables uniform cell distribution on the scaffold within the rotating seeding chambers, despite the complexity of the geometry^30,31^.

The use of brush motors, to make the sphere turn in a 360° random rotation at low speed, is associated with some limitations as it has reached the maximum of its capacity. Upgrading to servomotors would allow for a more precise rotation while eliminating the risk that the motors would stop on their own due to overheating, increased resistance and decrease in voltage. This rotary cell seeding system uses a potentiometer to regulate the voltage to the motors and to dictate the rotational speed. The addition of a microcontroller system and an electronic display would allow a better voltage adjustment and more precise rotating speed during cell seeding.

Compared to other seeding techniques, the developed spherical seeding system method allowed uniform cell distribution on PETG scaffolds as well as to produce thicker matured TEBV-A after 42 days of cell culture regardless of the number of cells initially adhered to the scaffolds during the seeding period. A more uniform cellular distribution may then help tissue maturation and consequently improve ECM secretion and assembly^32–34^. It has been shown that cell–cell and cell-ECM interactions in tissue engineering are needed in disease modeling to better represent crosstalk between cells, tissue compositions and the microenvironment associated to complex human pathologies^11,35,36^.

Though complete TEBV model incorporating an *adventitia* (fibroblasts), *media* (smooth muscle cells) and *intima tunica* (endothelial cells)*,* the current state of branched TEBV models is evolving rapidly from branched vascular prostheses to branched vascular stents in the clinic to branched collagen tubes with endothelial cells and to 3D-printed branched vascular grafts^4,37–39^. To the best of our knowledge, we are the first research team to produce an all biological small-caliber branched TEBV model of the *adventitia* without exogenous material. Of particular interest, the critical junction site remained intact and did not rip off following scaffold disassembly. This innovative system makes possible the production of tissues with a sufficient thickness allowing their manipulation, observation, and study with only one monolayered fibroblast sheet, although it cannot yet be perfused. Multiple sequential cell seedings could be the next appropriate step to make thicker multilayered TEBVs^40^.

The production of patient-derived small-caliber TEBVs with complex geometry may be an innovative way to model various vascular diseases. For instance, the constructed Y-shaped TEBVs would be a unique in vitro model to study intracranial aneurysms (IA), mainly occurring in the bifurcations of the Circle of Willis in affected individuals^41,42^. IA is a cerebrovascular disorder in which weakness in the wall of cerebral blood vessels causes a localized ballooning^43^. IAs present a 50% mortality rate and a 30–50% morbidity rate in survivors, following its rupture^44^. The use of Y-shaped TEBVs cultivated in a bioreactor allowing to generate artificial but physiological blood flow will be of particular interest in the study of IA and hemodynamics. Since the genetic component of IAs is still not very well understood, multilayered and branched TEBVs derived from patients to produce fully reconstituted blood vessels with *intima*, *media* and *adventitia* would also be of great interest for the study of pathogenic and pathophysiological IA-associated mechanisms.

In conclusion, we presented an innovating rotary cell seeding system that is easy-to-use, washable and sterilizable, allowing to produce five small-caliber tubular TEBVs of the *adventitia* with a complex geometry. Overall, the seeding system and chambers were conceptualized and custom-made to be of simple operation. The described system will therefore have considerable impacts for future research considering that evenly distributed cells all along the scaffolds during cell seeding is crucial for 3D culture and tissue engineering.

## Methods

### Rotary seeding system conception and construction

CREO 5.0 software (PTC, Boston, MA, USA) was used to produce CAD for the seeding system before construction (Fig. 1A). The sphere was made from two halves of a 10-inch diameter acrylic hollow sphere (California Quality Plastics, Ontario, CA, USA) (Fig. 1B,C). Two 3D-printed closure rings were made of PETG 1.75 mm filament (Filaments.ca, Mississauga, ON, Canada), printed on an H800 3D printer (Afinia, Chanhassen, Min, USA) and glued to the sphere halves with Silastic™ medical grade elastomer (Dupont, Wilmington, NC, USA). The closure rings are maintained together by stainless steel button head hex drive screws (McMaster-Carr, Chicago, IL, USA) screwed in the male PETG ring and locked in the female PETG ring. 3D-printed plates were conceptualized and printed as previously described to hold in place the seeding chambers. The plates were attached to the sphere with 3D printed supports that were glued to the center of the sphere half with Silastic™. To ensure the seeding chambers stay in place while the system is rotating, pressure was added via stainless steel corrosion-resistant springs (McMaster-Carr). The support plate was made from 6061-T6 aluminum alloy (Acier Picard, Levis, QC, Canada) using a 3-axis milling machine (Fryer Machine Systems, Patterson, NY, USA) (Fig. 1d). Two DC brush motors 12 V, 10 RPM (RobotShop, Mirabel, QC, Canada) were placed perpendicularly to one another, to provide the rotational movement for the system. Two motor brackets and three aluminum ball bearing-type supports were custom-made and attached to the plate. Corrosion-resistant stainless-steel balls were placed inside the support bearings to ensure proper rotation of the sphere and offer additional support (McMaster-Carr). The electronic components to supply current and control speed were placed inside a 3D printed PETG box. The motors were connected to separate 3 position switches, 25 k potentiometer, a linear voltage regulator (Digi-Key, Thief River Falls, Min, USA) and a medical grade AC/DC 5 V power supply (Newark, Mississauga, ON, Canada) that plugs in a regular 125 V wall outlet. All photographs and video were taken via iPhone 12 mini camera (Apple, Cupertino, CA, USA).

### Seeding chamber conception and construction

CREO 5.0 software (PTC) was also used to produce CAD for the seeding chambers (Fig. 2A–C). The chambers consisted of two distinct custom-made polycarbonate halves (Groupe PolyAlto, Quebec, QC, CA). Y-shaped indents were cut in both halves using the three-axis milling machine (Fryer Machine Systems). The bottom half was surrounded by a Y-shaped high temperature soft-silicone o-ring, 3/32 fractional width (McMaster-Carr) to seal the seeding chamber halves to one another (Fig. 2D). The top half was made with three openings that serve as seeding ports sealed off with a high temperature soft-silicone o-rings, 3/64 fractional width and 18-8 stainless steel cone-point set screws, M4 × 0.7 mm thread, 4 mm long (McMaster-Carr). The Y-shaped dismantlable scaffolds were custom-cut with a 5-axis milling machine (Fryer Machine Systems, Patterson, NY, USA) from 4.8 mm in diameter PETG rods (McMaster-Carr) and held together by 18-8 stainless steel dowel pins, 1/32" diameter, 1/4" long (McMaster-Carr). These scaffolds are placed in the center of the seeding chambers, inside the Y-shaped indent, and the two halves are joint together and are fully closed using 316 stainless steel button head hex drive screws, M4 × 0.7 mm thread, 16 mm long with general purpose 316 stainless steel washers for M4 screw Size, 4.300 mm ID, 8 mm OD and 316 stainless steel hex nuts, super-corrosion-resistant, M4 × 0.7 mm thread (McMaster-Carr) (Fig. 2E). All photographs were taken via iPhone 12 mini camera (Apple).

### Cell seeding parameter optimization

Human dermal fibroblasts were isolated as previously described^45^ and cultured in DMEM (Invitrogen, Burlington, ON, Canada) containing 10% fetal bovine serum (FBS; VWR, Radnor, PA, USA), 100 IU/mL penicillin G and 25 μg/mL gentamicin (Sigma-Aldrich, Kawasaki, OL, Japan). The use of human cells was approved by the ethical research board of the CHU de Quebec (Protocol number: 1115C & 1115D) and the individual was recruited on a voluntary basis following informed consent. Cells were seeded with 10 mL of DMEM culture media into the chambers containing 4.8 mm in diameter UV-C treated PETG rods (McMaster-Carr). Seeding chambers were placed in the rotary seeding system and kept at 37 °C for downstream experiments. UV-C treatment was performed as previously described for 30 min/side (90° turn) and then coated with 0.2% gelatin (Fisher Scientific, Waltham, MA, USA)^14^. To determine the optimal cell concentration to use during initial seeding three conditions were tested (0.10, 0.15 and 0.30 M/mL) and were added to the cell chambers, then placed in the rotary system at medium speed for 22 h. Three different rotating speeds were also tested to better optimize cell seeding parameters. Chambers were seeded with fibroblasts (0.15 M/mL), installed in the rotating sphere for 22 h at a rotating speed of 63°, 90° or 135°/minute, these speeds were considered to be the minimum, median and maximum reachable speeds by the sphere. Different incubation periods following cell seeding were also tested. Seeding chambers were seeded with fibroblasts (0.15 M/mL), placed in the sphere at a rotation speed of 90°/minute for 4, 8, 16 and 22 h.

### Cellular counts

Supernatants were recovered and centrifuged at 300×*g* for 10 min at the end of each of these incubation times. Remaining unattached and centrifuged cells present in the supernatants were resuspended in 1 mL of DMEM medium and counted using a cell counter (Beckman Coulter, Pasadena, CA, USA). Cell-seeded PETG rods or y-shaped scaffolds were first incubated with trypsin 0.05% (Fisher Scientific)/EDTA 0.01% (Teknisciences, Terrebonne, QC, Canada) for 10 min to let the cells detach, and then centrifuged at 300×*g* for 10 min. The recovered cells were resuspended in 1 mL of medium and then counted via Coulter cell counter (Beckman Coulter).

### Tissue-engineered blood vessel production

Human dermal fibroblasts were cultured in DMEM medium with 10% FBS and the antibiotic cocktail. 0.15 M/mL cells were seeded in custom-made seeding chambers via the seeding holes. A total of 10 mL of DMEM culture medium was injected in the seeding chambers containing Y-shaped scaffolds made from 4.8 mm diameter PETG rods (McMaster-Carr). PETG scaffolds were pretreated with UV-C and then coated with 0.2% gelatin (Fisher Scientific, Waltham, MA, USA) as previously described^14^. For spheric seeding, the seeded chambers were then placed in the sphere at 90°/minute for 22 h in a 37 °C room. For dynamic seeding, the seeded chambers were placed on an orbital shaker (BlotBoy, Benchmark Scientific, Sayreville, NJ, USA) for 22 h in a 37 °C, 8% CO_2_ incubator. For static seeding, the chambers were placed directly in a 37 °C, 8% CO_2_ incubator for the 22-h seeding period. Cell-seeded scaffolds were then recovered and placed in 500 cm^2^ cell culture plate containing DMEM culture medium, supplemented with 50 μg/mL ascorbic acid (Sigma), for 42 days in a 37 °C, 8% CO_2_ incubator. Medium was changed every 2–3 days and scaffolds were turned 180° each day of culture media changes.

### Tissue-engineered blood vessel analysis

Macroscopic images of cell-seeded Y-shaped PETG scaffolds were taken with iPhone 12 mini camera (Apple). Tissue thicknesses on the three different branches of the Y-shaped scaffold were measured with a high accuracy laser micrometer (Keyence, Mississauga, ON, Canada). Four different measurements on each of the branches of four different Y-shaped TEBVs were used for statistical analysis. TEBVs were fixed in 3.7% formalin overnight (ChapTec, Montreal, QC, Canada). Macroscopic photographs of the biopsied TEBVs junction sites were also taken prior to the histological analysis. Fixed 10 μm TEBV cross-sections were then stained with hematoxylin and eosin (H&E) as previously described^36^. Microscopic images were acquired and measured under bright-field conditions using an upright microscope (AxioImager.M2; Carl Zeiss Microscopy, Jena, TH, Germany). For distribution analysis, seeded scaffolds were fixed in 3.7% formalin for 30 min than placed in Rhodanile Blue stain for 15 min, then rinsed before letting dry before taking macroscopic photographs of all sides of the scaffold.

### Live/dead assay

After the 22-h seeding periods, supernatants were recovered and centrifuged at 300×*g* for 10 min for each seeding techniques. Cell-seeded PETG rods were incubated with trypsin 0.05% (Fisher Scientific)/EDTA 0.01% (Teknisciences) for 10 min to let the cells detach, and then centrifuged at 300×*g* for 10 min. The recovered cells from supernatant and scaffolds were incubated for 15 min with Calcein AM 2.5 nM (Thermo-Fisher) to label live cells and Ethidium homodimer-1 4 μM (Thermo-Fisher) to label dead. Cells were immediately analysed with a BD FACSMelody™ flow cytometer (BD Biosciences) and data were treated using the FlowJo™ v9 software (Ashland, OR, USA). For mature tissues, they were first recovered from the scaffolds and placed in a digestion solution of 5.7U/ml collagenase H (Sigma-Aldrich) in accutase (Sigma-Aldrich) at 37 °C with 300 rpm agitation for 30 min. Cells were filtered through a 40 μm cell strainer (Fisher Scientific) and centrifuged at 300×*g* for 10 min. Finally, cells were analysed as described below.

### Statistical analysis

Statistical analyses were performed using the GraphPad Prism 9.0 software (GraphPad, San Diego, CA, USA). Data presented in box and whiskers graphs with max and min were analyzed by two-way ANOVA with Tukey’s multiple comparison test or by Kruskal–Wallis test with Dunn’s multiple comparison test. Data presented in scatter plot with mean and standard deviation (SD) were analyzed by Kruskal–Wallis test with Dunn’s multiple comparison test or by Welch’s t test. Data presented by stacked bars with SD were analyzed by Kruskal–Wallis test with Dunn’s multiple comparison test. A *P*-value of < 0.05 was considered statistically significant.

### Institutional review board statement

The study was approved by our Institutional Ethics Committees (Ethical research board of the CHU de Québec; protocol number 1115 C & 1115 D. For more information, please contact (gurecherche@chuq.qc.ca). All experiments were performed in compliance with the national Tri-Council Policy Guidelines: Ethical Conduct for Research Involving Humans and approved by the ethics committee of the CHU de Quebec—Université Laval.

### Informed consent statement

Informed consent was obtained from all subjects involved on a voluntary basis in the study.

## Supplementary Information

Supplementary Figures.

Supplementary Video 1.

## Data Availability

The data presented in this study are available on request from the corresponding author.
