# Supplementary material for: Spherical rotary cell seeding system for production of small-caliber tissue-engineered blood vessels with complex geometry
Source: Sci Rep. 2023 Feb 21;13:3001. doi: 10.1038/s41598-023-29825-0 (PMC9944280; doi:10.1038/s41598-023-29825-0)
Supplement: Supplementary file 1 — Supplementary Figures. [file 41598_2023_29825_MOESM1_ESM.docx]

Supplementary Figures


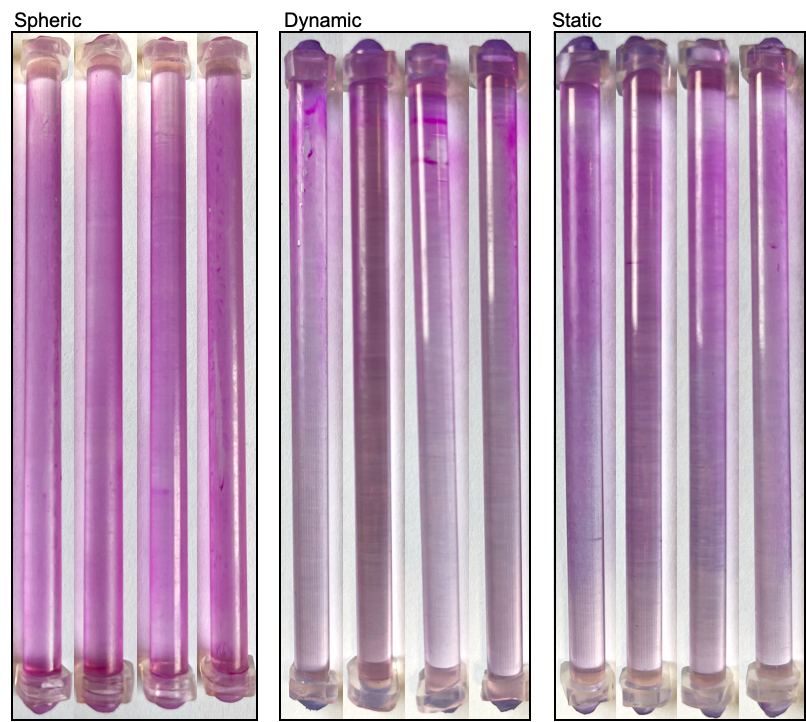


**Supplementary figure 1.** **Cellular distribution in post-seeded TEBV-A produced by different seeding techniques.** Photographs of Rhodanile Blue stained cells on PETG scaffold post-seeding.


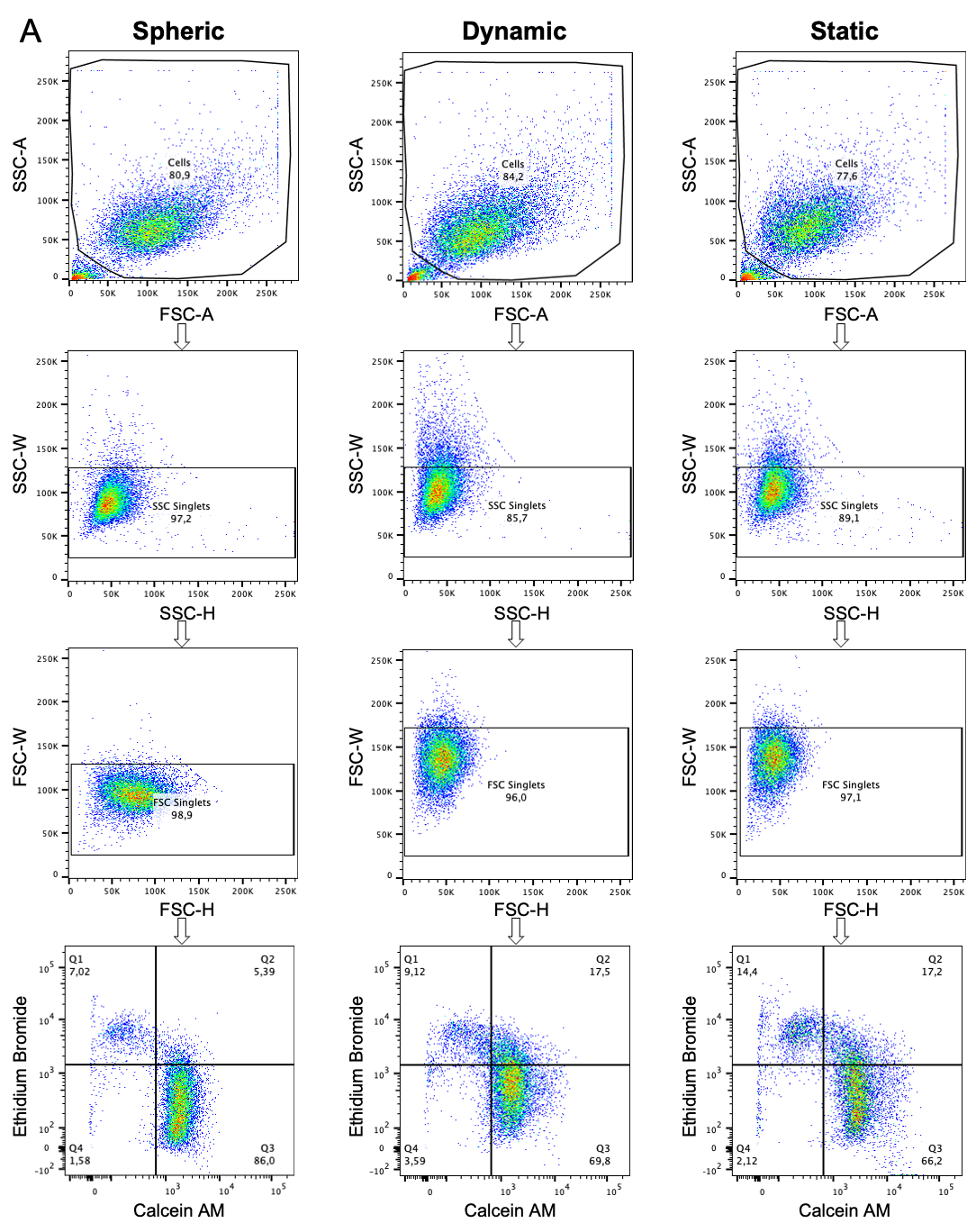


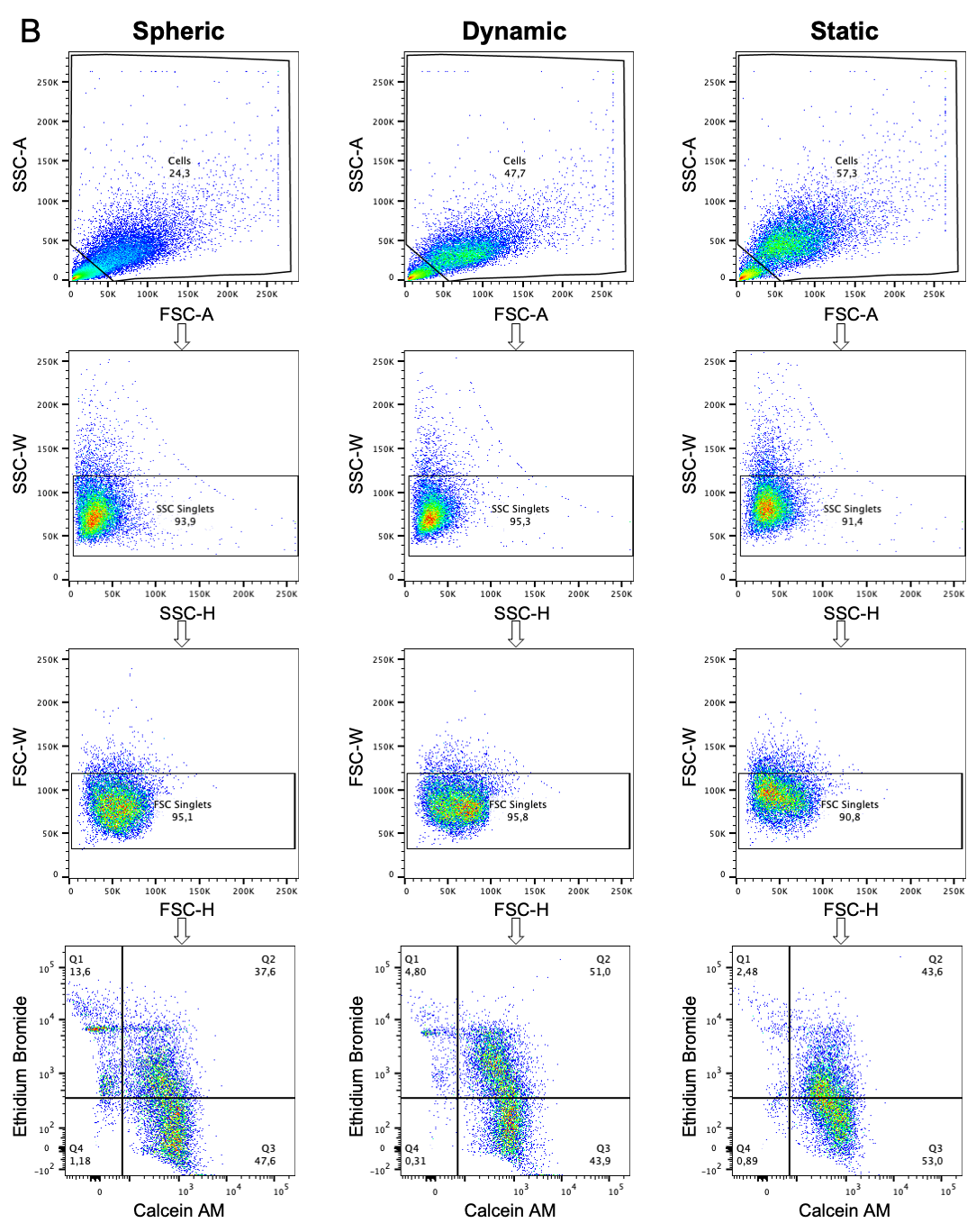


**Supplementary figure 2.** **Gating for flow cytometry analysis for Live/Dead Assay.** (**A**) Gating post-seeding and (**B**) post tissue maturation for different seeding techniques.
